# Small Ruminants: Farmers’ Hope in a World Threatened by Water Scarcity

**DOI:** 10.3390/ani9070456

**Published:** 2019-07-18

**Authors:** Oluwakamisi F. Akinmoladun, Voster Muchenje, Fabian N. Fon, Conference T. Mpendulo

**Affiliations:** 1 Department of Livestock and Pasture Science, Faculty of Science and Agriculture, University of Fort Hare, Private Bag X1314, Alice 5700, Eastern Cape, South Africa; 2 Department of Animal and Environmental Biology, Faculty of Science, Adekunle Ajasin University, Akungba-Akoko PMB 001, Ondo-State, Nigeria; 3 Department of Agriculture, University of Zululand, Kwadlangezwa 3900, P.O. Box 3886, Kwazulu-Natal, South Africa

**Keywords:** sheep, goat, water restriction, adaptation, thermoregulation, water scarcity

## Abstract

**Simple Summary:**

Water is one of the most important nutrients to livestock. It is so essential that more than half the volume of the animal's body is water. However, its availability is threatened by the irreversible changes in climate, which has culminated into reduced rainfall in most regions of the world. Such an increasing threat to regular water supply, and by extension to food security and livelihood has forced a shift from large to small ruminant production, especially in regions experiencing low rainfall, with farmers taking advantage of their adaptive process and efficiency of water use. Small ruminants, especially desert goats, can adaptively survive in water-limited areas while trekking long distance in search of feed and they will regain any weight loss at the next watering point. Further research is needed on adaptive indigenous breeds of small ruminants since tolerance to water scarcity is breed dependent, so that improvements can be made through effective selection and breeding program.

**Abstract:**

The availability and sustainability of suitable and good quality drinking water is a global concern. Such uncertainties threaten livestock production with an attendant ripple effect on food security. Small ruminants, including sheep and goats, appear to be promising to smallholder farmers in solving this problem because of their ability to survive in water-limited areas and harsh environment when compared with large ruminants. Their small body size is also seen as an advantage, because less water will be required for proper digestion and feed utilization. Therefore, this review will provide information regarding the adaptive responses of small ruminants on thermoregulation, blood metabolites, immune status, drug pharmacokinetics, reproduction and hormonal indices during the period of water stress. Adaptable and indigenous breeds are known to be more tolerant to water stress than selected breeds. A drop in feed intake and weight reduced respiratory rate and increased concentration of blood metabolites are the general effects and/or observations that are encountered by small ruminants during the period of water stress. The concept of water tolerance either as deprivation and/or restriction of indigenous and adaptable breeds of small ruminants is gaining ground in research studies around the world. However, more research, however, seeking to explore water tolerance capacity of adaptable breeds especially in arid and water limiting areas are still needed.

## 1. Introduction

Over 40 per cent of the people on earth surface is already affected by the scarcity of fresh water and more than 1.7 billion people are currently living in a highly water-limited basin [1]. Demand for water will continue to rise across the globe as a result of climate change, increased demand from rising population, polluted supplies, land use and economic change [2]. The Fourth Assessment Report that emanates from the Intergovernmental Panel on Climate Change [3] affirms that the stress on future water resources will continue to increase if mitigating strategies are not put in place. Areas that are most vulnerable and with very limited freshwater resources are the North Africa, Northeast China, Pakistan, India, North America and Middle East [4,5]. Presently, the inability to satisfy the demand for good quality water has reached a critical stage in many areas of the world. Such an imbalance creates concern in terms of meeting up with the water-requirements for food production for the growing population of the world which, based on projection, will reach 9.8 billion by the year 2050 [6]. 

Water is an important nutrient and it should be readily available for animals in the required amounts [7]. However, livestock is the worst hit with the unavailability of suitable drinking water becoming an enormous concern for farmers. Low rainfall affects drinking water and reduces the availability of feeds for small ruminants, especially for those that are grazing in semi-arid zones [8]. The unavailability or insufficient amount of drinking water for livestock negatively impact on all performance indices and it may result in food insecurity, especially for smallholder farmers in developing countries.

The way forward under this shifting condition is probably to decide the animal species (non-ruminants, large or small ruminants) to rear and produce. In this regard, small ruminants appear to be more promising because of their low production cost, short generation interval, suitability to small holdings, multipurpose (meat, milk and fibre) use, ability to utilize crop residues effectively, and most importantly their tolerance to harsh climatic variables (such as low rainfall and heat stress) than cattle and other monogastrics. The shifting trend of keeping more small ruminants than cattle by pastoralist (the Samburu) in Maasai, Kenya and Afar, Ethiopia was partly because of their drought-tolerant ability [9]. Peacock [9] argued that increased frequencies of drought have resulted in the inability of pastoral families to re-establish larger stock as a result of the constant struggle to ‘recover’ from the last drought. For this reason, they increasingly depend on small ruminants. In addition, the preponderance of small ruminants, especially sheep and goats over cattle and other animal species in Asia and the Middle East (low rainfall areas) that are currently faced with desertification and high temperature [10], is indicative of their drought-tolerant abilities. Seo and Mendelsohn [11] estimated that the probability of choosing beef and dairy cattle decreases rapidly at elevated temperature as compared to a higher probability of choosing goats and sheep in a graphical illustration on the choice of livestock species with respect to drought-induced temperature. However, the choice of chicken is however estimated to assume a normal distribution curve (bell-shape) with a maximum peak at mean temperature of 22 °C. 

Many research studies have shown the capability of goats [12] and sheep [13,14] to tolerate dehydration. Small ruminants are imbued with water saving mechanisms (e.g. reduced panting and respiration rate) that help them to minimize the loss of water and thus enhance their ability to withstand water shortage [15,16]. They have evolved an efficient water economy and this has enabled them to maintain their intake of dry matter and production during times of water shortfall or scarcity, even at elevated temperature. This is because, dry matter digestibility is improved and coupled with a reduced metabolic rate during dehydration. In addition, the rumen also performs the function of a water reservoir (15% of animal body weight) for use when water is scarce [12]. Such adaptation ensures that they continue to live in spite of extended periods of water scarcity while grazing several kilometres away from points of water and efficiently exploiting the sparsely distributed pastures. During water stress, small ruminants respond by reducing their feed intake culminating in weight reduction as a result of body mass and water loss [17]. Water losses amounting to 18% of body weight can be tolerated by cattle; sheep and goat, 20%; camel, 25% and even more than 40% in some desert Bedouin and Barmer goats because the fore-stomach can accumulate water to be used when supply is low. However, a body mass loss of 15% and above due to reduced water intake in other animals is deleterious and can be fatal [18]. Although such an imbalance in water and energy metabolism produces a negative impact on general health and productivity, small ruminants, including sheep and goat, have evolved adaptive mechanisms that enable them to successfully thrive and breed in water-limited and arid lands. Therefore, the present review, seeks to discuss water resources and use in a future perspective, current small ruminants distribution, the impact of water scarcity on these animals and the adaptive mechanisms that are evolved by them.

## 2. Global Water Resources and Use 

**Figure 1:**
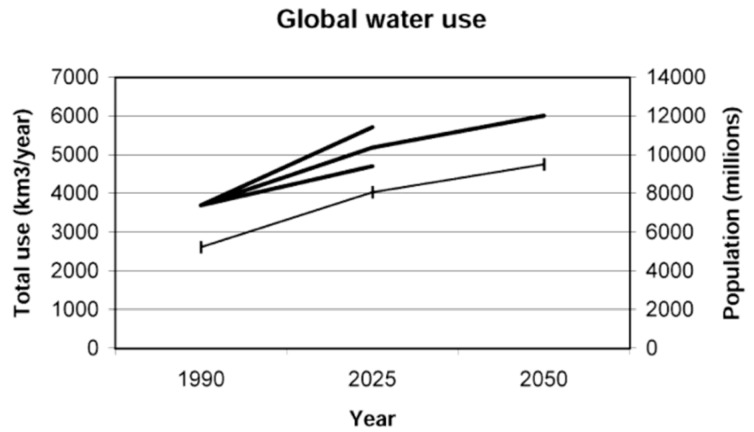
Global water resource use. Source: [25].

The percentage of fresh water from the total volume of water on Earth is estimated to be about 2.50%. However, about two-thirds of this is locked in glaciers and ice caps [19]. Just 0.77% of all water (~10,665,000 km^3^) is held in aquifers, soil pores, lakes, swamps, rivers, plant life, and the atmosphere [19]. The mean annual surface and subsurface (groundwater/shallow aquifer) runoff that accumulated as river discharge is assumed to constitute the sustainable water supply that is accessible to local human populations [20]. According to Kundzewicz and Döll, [21], about three billion people source their drinking water from groundwater. Unfortunately, most of the fresh and groundwater resources accessible to the population have been largely depleted, consequently resulting in a sharp drop in agricultural production and inflated prices. In arid and water-limited regions, the expected precipitation over the next century will decrease by 20% or more and the situation may be even worse with an unprecedented increase in population. Unfortunately, these regions’ (Asia, Arab, Middle East, Northeast China, India and some Africa countries) economy largely depends on natural resources and climate-driven activities [22]. Fresh water-limited areas will experience the largest percentage and absolute increase in demand-driven water stress, with agriculture remaining as the biggest user based on the conventionally developed scenario (CDS) of projected water use developed from conjectures regarding probable alterations in the components of demand [4,5]. It was estimated that by the year 2025, over 30 countries will be found to be water stressed as compared to seven countries in 1955, and by the year 2050, two-thirds of the world population may be already water-stressed simply due to a rise in population, industrialization, global climate change and water use [23]. Climatic changes, population growth and clustering, irrigation expansion, industrial revolutionary changes, the efficiency of water use and demand management will influence future water resource and use [24]. Figure 1 is a projection of global future water resource use (1999–2050).

As estimated recently [26], animal agriculture utilizes about 8% of the available global water supply. Out of this, about 1% is available for animals’ nourishment, servicing on-farm activities and the processing of animal products into food. The remaining 7% is used in irrigating feed crops, in intensive production of livestock in developed countries. Given the above projections of high water demand and use in agriculture, all of the procedures in the livestock sector involving water use during production and management must be reviewed. Information regarding distribution patterns and feeding habits, adaptation to climatic changes especially to limited water must be critically reviewed and updated for their proper management and sustainability of the livestock industry.

## 3.Small Ruminant Distribution 

Small ruminant agriculture plays an important role in the social and economic development, especially for developing countries. They contribute to the management and development of landscapes, ecosystems maintenance, biodiversity conservation and provision of job opportunities from their products (meat, skin, milk etc.) and by-products in the market [27]. The sector is so important such that 56 per cent of the world ruminant domestic populations (3872 million heads) are sheep (1178 million) and goats (1000 million) [28] and they are distributed all over different types of ecology. Over 56 per cent of the world's small ruminants are located in water-limiting and dry zones in developing countries, whereas temperate and humid zones account for 27 per cent and 21 per cent, respectively [27,28]. Though less than sheep and cattle, the goat population of the world has constantly increased since the 60 s especially in the countries of low income or less favoured regions of the world [29], and a 60% increase in global sheep number is expected by 2050 [30]. According to the working document of Scherf [31] that was submitted to Food and Agricultural Organization (FAO) on domestic animal biodiversity, sheep and goats, clustered in 1314 and 570 breeds respectively, and are distributed across various geographical and agro-ecological zones around the world. Table 1 shows the population size and distribution of buffalo, cattle, sheep and goat around the globe excluding extinct breed. Market demand and shifting to more profitable agricultural activities seem to be the main determinants for changes in the small ruminant population [32]. This small ruminant sector (sheep and goats) contributes about 25.6 million tons of milk and 1.5 million tons of meat annually [28]. The majority of the grazing lands around the world are found in seasonal environments with marked variations in resource abundance, with the arid and semi-arid zones of the tropical belt being characteristics examples. 

The socio-economic importance of small ruminants (especially sheep and goat) both to smallholder and commercial farmers in the entire world cannot be overemphasized. This is because the demand for their product and by-products' keep increasing with population increase. Therefore, there is a need to continue investigating the different methods of sustaining the increase in demand with limited population resources. With the limitation of fresh water as observed in the previous section, there is a need to understand the water requirements of these animals so that their production can be sustained and effectively managed.

## 4. Water Requirements and Metabolism 

The amount of water that is required by small ruminants is a function of their body metabolism, ambient temperature, body size and weight, restriction patterns, dry matter intake, feed composition and energy consumed, water quality, species, physiological status, production stages, breed and wool covering [33]. The amount of water that is voluntarily consumed by ruminants is two times that of dry matter consumption. Water consumption tends to increase when diets that are rich in protein or salt are fed to animals. However, the expression of water requirement per animal is usually the total of the needed amount for all physiological stages (i.e., maintenance, growth, pregnancy and lactation) [33,34]. According to Beede [35], satisfying the daily requirement is fulfilled when the net water intake to water loss is zero [i.e., (free drinking water + water in or on feeds consumed) = (water excreted in urine and faeces + water secreted in milk, sweat and respiratory pore)]. In the view of Esminger et al. [36], the actual total water that is required by ruminants is a complex process and a balance must be struck to ensure that the total water intake (TWI) equals water loss (WL) and water retained (WR). TWI that is accessible to animals includes water sources from metabolic (nutrient catabolism), drinking and feed water. Nutrient catabolism often generates some metabolic water as part of the end product, which is also available as a water source to the animal. Based on that assumption, 1g of metabolized carbohydrates, fat and protein will yield 0.56, 1.07 and 0.42 g of water, respectively [37]. However, higher water losses by the lungs due to an increase in breathing in the case of fat oxidation usually result in the production of less metabolic water during fat hydrolysis when compared to carbohydrate hydrolysis [36]. In addition, water concentrations in succulent feeds like fresh legumes, grasses and silage are usually very high, constituting an important source of water for sheep and goats that are reared in arid and water-limited areas [38]. 

Water intake per animal depends on the degree of dehydration or restriction, drinking time allowances, and the number of animals drinking together at a particular watering point [35]. Therefore, mammals were grouped based on their ability to rapidly or gradually replenish lost water. Sheep and goat can drink and adequately replace 18–40% of their body mass within 3–10 minutes at first drinking [12]. During heat stress, water intake increases while the feed consumed decreases and weight gain decline, a situation that is parallel to feed consumption and nutrient balance [39]. In addition, the attempt to balance body temperature by an animal during elevated temperature often results in increasing their energy [40]. The way that drinking troughs are arranged and the ease of accessibility of animals to points of water supply also affect water intake [38]. This is a common observation during the dry season especially in arid and water-limited areas when the points of water supply get limited and accessibility decreases.

As pointed out, more than half of the small ruminant population in the world is found in water limiting and arid regions. Therefore, there exists, a possibility of an adaptive response that favours their survival, growth and reproduction.

## 5. Adaptive Responses of Small Ruminants to Water Shortages and Deprivation

Severe water shortages for herds are very common in the arid and semi-arid regions around the world. The poor forage quality and low humidity level that usually accompany the dry season period further compound this. Water intake by an animal is usually restricted to once per day, during access to the water source [15]. Research studies on small ruminants simulating conditions of water scarcity or limitation in arid or water limiting areas in the form of water deprivation and/or restriction with a view of assessing their adaptive responses or changes at all levels of production and physiology are gaining global attention.

### 5.1. Effect on Metabolic, Rectal Temperature, Respiratory and Pulse Rate

Evidence abounds that the metabolic rate in animal decreases during water restriction, a process that is suggestive of an energy conservation response [41]. Such imbalance in water intake can precipitate an increase in body heat and rectal temperature [42,43]. According to Davis and DeNardo [44], an adjustment to a lower metabolic rate and slower water loss during the period of dehydration enhanced the survival value of an animal because the duration that an animal can survive without eating under dehydration is extended. Evaporative cooling via sweating which constitutes a major cooling avenue during heat stress for goats adapted to the hot arid zone [39] is usually reduced in dehydrated animals [45]. Dehydration-induced hyperthermia may be adaptive in conserving water, as it increases the temperature at which animals switch from thermoregulation via convection and radiation to evaporative cooling [46]. The rectal temperature (RT) of Nubian goats significantly increased in the third day of water deprivation, despite the marked decrease in feed intake that could have influenced the energy budget. The daily average rectal temperature in dehydrated goats was 0.5°C to 0.9°C higher than the hydrated ones, a pointer to a reduction in evaporative heat loss [47]. RT increases as water deprivation continues in the three local Saudi Arabia goat breeds. However, in lactating and dry Awassi ewes that were watered once every three days or daily, no changes were observed in their RT [48]. A similar result was observed in water restricted (W80 and W60% of *ad lib* water) Lacauna ewes [49] and Aardi goat [50]. Sheep are noted to be thermo-stable even during periods of dehydration [51]. When water was deprived, there is a rise in body temperature of small ruminants as a result of their reduced their thermoregulatory evaporation [52]. This depression of cutaneous evaporation presumably triggers the water conservation mechanism [45], with an attendant rise in RT during the water deprivation period. Respiratory activity was noted to be reduced during periods of water deprivation in goats [53] and sheep [54]. Table 2 summarizes the outcomes from experimental studies on changes in rectal temperature following intermittent watering regimen. Changes sometimes in respiratory rate (RR) seem undetectable in goats [55] due to the combined effect of heat stress and water deprivation. Water restricted Lacauna ewes (W80 and W60% of *ad lib* water) had their respiratory acts per minute reduced from Day 0 to Day 14. As a defense mechanism, small ruminant reduces respiratory activities during the period of water deprivation to prevent water loss and dehydration via pulmonary evaporation [49]. The water conservation mechanism in small ruminants ensures that water losses via respiration are effectively managed, during shortages. The panting rate (breathe/min) decrease in water restricted and dehydrated sheep at an elevated temperature [43]. However, RR could increase during periods of water shortages and elevated temperature. Table 3 shows the outcome of the different experiment following intermittent watering on the respiratory rate.

### 5.2. Drinking Behaviour, Body Weight and Feed Intake

Tolerance to water scarcity or dehydration can be judged based on changes in body weight during the period of water deprivation. Giving the close relationship between body water and weight, water-tolerant animals are those that possess the ability to conserve water more [54]. Small ruminants have the tendency to recoup as much water as possible in the rumen during rehydration, an adaptation mechanism that allows for them to endure severe dehydration. This high rumen volume usually exceeds the extracellular fluid volume causing a sudden drop in rumen osmolality and a huge osmotic gradient (200–300 mOsm/kg) between the rumen and systemic fluid [12]. Ethiopian Somali water-restricted goats drank 1.34 (watered every second day), 2.01 (watered every third day) and 2.51 (watered every fourth day) times as much as the water *ad lib* group [56]. During rehydration, the Bedouin goats were also reported to consume large volumes of water [57]. However, the average daily water intake decreases when the water deprivation exceeds 48 h. Rams watered at an interval of 24, 48 and 72 h had reduced water intake when compared with those that had free access to water [58]. When Tswana goats were water deprived for 48 and 72 h, their free water intake dropped as compared to goats watered every 24 h [59]. Water deprivation usually results in body weight fall and it becomes more pronounced when the ambient temperature is high especially during summer [12]. Sheep that were watered only in the evening (20:00 p.m.) had their body weight reduced by 7.00% (winter) and 11.00% (summer) [60]. In a study that was conducted by Alamer and Al-hozab [61] on the effect of water deprivation and season on body weight changes in Awassi and Najdi sheep, it was recorded that in spring, body weight decreased by 13.30% and 15.00% in Awassi and Nadji sheep, respectively, whereas 18.00% and 21.50% decreases were observed during summer. In a three days water deprivation study at elevated temperature using three local Saudi Arabia goat breeds (Hipsi, Aardi and Zimri) their body weights were reduced by 21.00% [53], as opposed to Sudanese male goats, which under the same period of water deprivation showed 18.00% of body weight loss at a lower ambient temperature [62]. Such higher body loss at elevated temperatures is attributed to water losses via the respiratory and cutaneous routes. The loss of body weight connected with water shortages can be attributed to feed and water intake reductions, coupled with body water loss [63].

The need to compensate for a decrease in dietary intake, leading to the mobilization of fat (and possibly muscle) for energy metabolism [13], produces weight loss. However, such weight loss is quickly regained at the next watering point. A 16.30% loss in the body weights of Dorper sheep was replenished in just a few minutes of water availability [67]. The replenishment of all water losses by the black Bedouin goats was accomplished in a couple of minutes of rehydration [68]. However, breed, species, rumen capacity and the immensity of weight loss affect the speed with which body weight can be replenished. Marked responses to feed intake during water deficiency under various water restriction regimens were detected in Yankasa sheep and native goats [58,69]. In a water restriction experiments that were conducted at an ambient temperature of 22°C, Maloiy et al. [70] reported an increasing and significant reduction in feed intake from Turkana goats (−58.30%) to fat-tailed sheep (−48.00%) and Zebu (−50.00%). However, some studies reported that feed intake is not significantly affected by water restriction [20,71], because the ruminants can store up water for use during scarcity or shortfalls. According to Hadjigeorgiou et al. [72], dry matter voluntary intake in sheep water-restricted for 1h per day or given 65% of the *ad libitum* intake of water was not affected. In contrast, others have observed a significant decline in feed intake following water deprivation. For example, when the sheep were water deprived for three days, their feed intake was significantly reduced [61], and the Nubian goats reduced their feed intake by about 60.00% during the first day of water deprivation and consumed only 5.00% of the control intake by the third day of water deprivation [8]. After three days of water deprivation, Egyptian Baladi goats were able to maintain only about 35.00% of the control feed intake [73], while Aardi goats deprived of water for 2 days almost stopped eating completely [74]. However, a comparative study between camels and goats revealed a higher and significant decrease in roughage feed intake for camel (−54.60%) as compared with goats (−27.80%) following a 72 h of water deprivation [75]. In a similar comparative study, Mousa et al. [76] observed that water restriction in sheep, goats and camels for five days caused a decrease in dry matter intake in the three species, but the reduction was higher in camels than in the other two species. This further explains their preference of rearing sheep and goats over other animal types during the period of drought and water scarcity. 

The type of feed that is offered to an animal during water restriction also affects feed intake. Goat fed legume hay had their feed intake reduced by 18.80% as compared to a reduction of 21.20% when a low protein content diet (meadow hay) was fed [77]. Similarly, Osman and Fadlalla [78] used eight adult water-restricted rams in five successive trials, which were fed different feeds (*Medicago sativa* hay, *Doclichos lablab* hay, *Zeamais* hay, concentrate mixture and a mixture of dry desert grasses) and observed that animals that were fed the desert grass mixture had significantly lower dry matter intake (34.17%) than those that were fed Lucerne hay (8.00%). It has also been shown that Bedouin goats consumed more lucerne hay than wheat straw during dehydration [79]. 

Drinking is shown to be positively correlated with feed intake in ruminants, and therefore, the adaptive mechanism during the period of water shortage that leads to reduced dry matter intake would help to further reduce the water loss that is linked with feed metabolism and heat dissipation [80]. Small ruminants seem to be more resilient to dehydration than cattle. In fact, sheep that are acclimatized to arid tropical weather conditions may be expected to survive for 6–10days without drinking water, while cattle under range management in desert areas died following 3–5 days without water [81]. When compared with sheep, cattle had shorter survival time in during water deprivation, which was linked to a greater rate of water losses than sheep. In addition, the ability to effectively concentrate urine by sheep and/or goats makes them superior to cattle in terms of adaptability to water-limited areas and arid environments [17]. A reduction in feed intake during water restriction could be possibly induced by the reduction of postprandial hyperosmolality of the ruminal fluid [82]. The decrease in the volume of the circulating blood (hypovolemia) and high blood solute concentration (hyperosmolality) may occur after feed intake in the animal because of the secretion of saliva and gastric juices, a mechanism forcing them to drink while eating, or on the other hand reduce feed intake during water restriction [16]. 

### 5.3. Effect on Nitrogen Balance and Digestibility

Water restriction improves the digestibility of nutrients by increasing digesta retention time to allow more time for degradation by microbes and microbial synthesis [14,78]. Corriedale ewes that were water restricted for 2 and 3h after feeding had greater digestibility when compared to those that have access to water *ad libitum* after feeding [7]. Although both rumination and digestion require water, the digestive capacity of the rumen is enhanced during water restriction, resulting in an improvement in feed digestibility. Positive nitrogen retention in water-restricted sheep increased the crude protein digestibility when compared with the negative nitrogen retention in sheep with access to *ad libitum* water after feeding [7]. A decrease in urine output and nitrogen losses is associated with water deprivation or restriction, and this could probably be the result of reduced filtration in the kidney glomeruli that was produced by water deprivation [7,83].

### 5.4. Effect on Blood Metabolites

Kumar et al. [64] restricted the water of Malpura ewes at alternate day, 20% and 40% of *ad libitum* water and reported that the 40% treatment group had higher haemoglobin (Hb) and Packed cell volume (PCV), with the ‘alternate day’ group being significantly lower. A similar result of high haemoglobin concentration was reported in sheep that were water restricted for four days [60]. Increases in Hb and PCV values following water restriction have been attributed to severe haemoconcentration due to reduced water intake. However, local goats were less affected, as shown in a three days comparative study with water deprivation, as reflected in their low PCV (19.33%) and Hb (7.43%) when compared to 34.00% and 15.97% in camel’s PCV and Hb respectively [75]. Due to low feed intake following water restriction, plasma glucose in sheep drops [60] below the normal range of 48–75 mg/dL [84]. Plasma glucose concentration in response to water restriction in goats remains unchanged as reported by some authors [53]. However, other studies reported a decline of 13.00% in the plasma glucose level in Sudanese desert sheep watered every 72 h [58]. Insufficient water intake increasestheconcentrations of creatinine, cholesterol and total protein and plasma glucose in Yankasa and Awassi sheep [13,85]. There were changes in the cholesterol, creatinine, total protein, albumin, urea, sodium, chloride and triglycerides in Comisana ewes restricted of water at 60% and 80% of *ad libitum* water intake for 40 days [14] as a result of haemoconcentration phenomena caused by a lower blood water level. The increase in the concentration of blood metabolites (cholesterol, urea, creatinine, total proteins and electrolytes) was also confirmed in several studies in different sheep [7,13] and goat [53] breeds that were subjected to water restriction. High blood urea concentration following water restriction was as a result of the kidney taking up much water and with reduced blood flow towards the urinary apparatus [49] while elevated creatinine was attributed to changes in the clearance rate of endogenous creatinine [86]. An increase in sodium concentration in water restricted animal is due to a greater aldosterone activity that increases the electrolyte level in the kidney and gradually declines probably due to a decrease in feed intake and volatile fatty acid production in the rumen [87]. Cl^−^ concentration follows the same pattern as Na^+^ since Cl^−^ is passively distributed according to electrical gradients that were established by the active transport of Na^+^ [88]. Studies are divided on concentrations of Ca^++^ and K^+^ under water restriction. Some reported increasing levels [14,49] while others reported no difference in blood K^+^ following water deprivation [48,85]. The plasma potassium concentration in water restricted Awassi sheep (watered once every four days) was reported to decrease [13]. However, pregnant Yankassa sheep that were watered once every 48 or 72 h had increased potassium concentration but the concentration remains unchanged in non-pregnant Yankasa sheep [85]. Plasma osmolality generally increases with an increase in plasma sodium concentration following water deprivation and drops to low values the day after drinking. This is due to the fact that sodium and its associated ions majorly determine osmolality and extracellular fluid volume [56].

### 5.5. Effect on Physiological Status 

#### 5.5.1. Effect on Reproductive Traits and Hormones 

Adequate supply and availability of sufficient water resources are required both for both survival and reproduction. Hence, any imbalance or shortfall of water resources below the optimal level can compromise an animal's health and general vigour [89]. Dehydration at elevated ambient temperature decreases the plasma volume as a result of the uptake of water by the tissue and an attempt by the animal to maintain fluid balance usually results in increased secretion of aldosterone and cortisol [90]. Usually, the endocrine glands are stimulated following water restriction with the sole purpose of modifying the metabolic activities depending on the ambient temperature [91]. Alteration in follicular growth and by extension, reduced oestrus cycle, has been linked to negative energy balance and reduced feed intake resulting from water deprivation [92]. Oestrus duration in Kivircik sheep in the semi-arid region was reduced when feed intake drops (30.00% of *ad libitum* intake) [93]. However, when Malpura ewes that were treated with progesterone impregnated intravaginal sponges for estrus synchronization were water restricted (20%, 40% less *ad libitum* water and alternate day), the oestrus per cent and oestrus duration were not affected [64]. Nutritional insufficiency in small ruminants is usually implicated during periods of water scarcity evidently illustrated by their reduced feed intake and body weight. The effect is a decreased estrus response and estrus duration which is attributed to the delayed ovarian follicular maturation and impaired reproductive endocrinology [94,95]. Ovarian follicular development diminished during periods of stress as a result of repressed peripheral gonadotropins levels [96]. This became very noticeable in the water restricted groups of Malpura ewes [64] and undernourished ewes [97] having decreased plasma estradiol levels. Generally and during stress, productive functions such as growth and reproduction are suppressed by endocrine hormones in favour of survival and maintenance [98]. During periods of water scarcity or deprivation, the hormones are mobilized, giving their critical roles, to ensure that the energy needs are satisfied and water losses minimized. Fat mobilization is conjoined and modulated by a decrease in insulin levels due to a decrease in feed intake in sheep [99]. Likewise, the leptin levels are decreased to guard against excessive mobilization in underfed ruminants, which if not curtailed could result in a high build-up of harmful circulating fatty acids [100]. This is because severe dehydration leads to a reduction in tissue perfusion and it can predispose animals to increased production of lactic acid and the development of lactic acid acidosis [101]. However, feed intake and plasma progesterone level are inversely related and this might be due to the differences in the metabolic clearance rate of progesterone rather than differences in secretion levels [102]. When the ewes were water restricted their plasma progesterone levels reportedly increase, a condition that is linked to reduced feed intake [64]. Plasma vasopressin (anti-diuretic hormone) concentration in water restricted animals usually increases above the maximum concentration and decreases following rehydration [56]. Kaliber et al. [103] water restricted (56, 73 and 87% of *ad lib*) 20 cross-bred and three-year-old female goats and reported an increasing trend of vasopressin with increasing restriction. Such higher levels of vasopressin during reduced body fluids balance help to maintain body water alongside extracellular fluid concentrations of sodium ion [104].

#### 5.5.2. Lactation

When lactating sheep and goat are water deprived for 72 h, milk production is affected in 50% of the sheep, leading to an increase in the viscidity of milk as well as lactose, fat, protein, fat and mineral salts [105]. On contrary, Casamassima et al. [14] water restricted Comisana ewes (100%, 80% and 60% of *ad libitum* water intake) for 40 days and reported that the quantity of milk was not affected. The author observed that the low water requirement precipitated by the low ambient temperature (6.1 °C) was generally the cause of the loss in milk production. At low temperature (5 °C), the blood flow towards the udder decreases, leading to a decrease in the prolactin secretion and consequently a reduction in milk production [106]. The black Bedouin goat is a desert-adapted breed and it has developed a high resistance to water scarcity as evidenced by its ability to sustain the production of milk for two days without water. When water was deprived for four days followed by two days of rehydration, the total milk yield and milk solids were about 70.00% of normal yields and normal growth of the progeny was not compromised [107]. In the water restriction study of Alamer, [52] the rate of drop in milk yield with 25% restriction was slightly higher than that with 50% restriction (20.00% vs. 18.00%). This indicates that water was effectively used in the group with 50% water restriction. The author attributed the drop in daily hay intake as the cause for the reported drop in milk solid and fat in goats with 25% restriction (as compared to a 50% restriction). A decline in feed intake following water deprivation/restriction is partly responsible for a decline in milk yield. During the stressful conditions (e.g., water deprivation), milk production was proposed to be down-regulated as a result of a reduction in mammary blood flow following a sustained period of dehydration [106]. The increased activity of plasmin (milk synthesis inhibitor) during water restriction is correlated with the fall in milk production. This modification in milk production enhances the survival potential in response to intense heat or water stress and the drop in yield is recovered following rehydration at the next watering point [108]. The Osmo-active milk component (urea, sodium and chloride) also rises with the intensity of water deprivation [16]. The lactating animals readjust to minimize losses in weight during a sustained period of water deprivation. In a six-day water deprivation study (50 and 25% water intake) live weight losses of lactating Aardi goats were stabilized not until after four days of water deprivation and thereafter [52]. This adjustment to sustained water restriction has been linked to the activation of and continuous increase in vasopressin (a water saving mechanism) which help to reduce the renal water secretion following a prolonged period of water dehydration in lactating goats [56]. Feed consumption dropped by 10.00% when lactating Moroccan goats were water deprived for two days [109]. However, feed consumption in Black Bedouin goats was maintained unchanged for 48h during complete water deprivation in [107]. The feed intake of lactating sheep receiving 60% of their normal water intake was also not affected during periods of lower ambient temperature [14]. This ability to maintain feed intake during the period of water shortages during lactation has been reported to be an adaptive mechanism with a view of sustaining an adequate supply of milk to nourish the newly born animals [52].

The lactating ewes had significantly reduced PCV and Hb concentration [48], as opposed to the usual haemoconcentration of blood metabolites in dry animals following water deprivation or restriction [60,64]. Similarly, haemoglobin formation was reduced in Barki ewes lactating for four weeks [110]. This is not surprising since the higher body water content and plasma volumes of lactating ewes due to increased water mobilization to the mammary glands could be responsible for the haemodilution [111]. Others obtained a similar result for total protein concentration, globulin, creatinine and urea in lactating ewes [48,110].

### 5.6. Immune Response and Drug Pharmacokinetics

A variety of biological functions are affected when an animal is deprived access to sufficient drinking water with respect to time. Water imbalance (restriction/deprivation) imposes stressful conditions on animal and negatively impact on their productivity and changesblood metabolites [48] including perturbations in behaviour and physiology [112]. A detailed analysis of the inter-relationship of deprivation, immune function and stress is yet to be conducted within a single species despite the negative correlations that exist between stress and immunity. Water imbalance is viewed as a physiological stressor that is capable of eliciting various endocrine responses [113]. During dehydration, the plasma glucocorticoids level, which is the principal hormone involved in stress response, is raised [114]. Such elevations may initially be beneficial on immunity, but if prolonged, they have more depressing effects, although the response may also depend on stress hormone concentration. This is as a result of its depressive effect on the synthesis or release of immune-promoting molecules and its potential to stimulate or depress the proliferation of B- and T-cells, depending on the physiological conditions [115]. However, a glucocorticoid response to water deprivation and/or dehydration is lacking in Awassi sheep [13,48]. Similar stressors either environmental or temperature related have the potential to alter immune parameters. Immunoglobulins (e.g., IgG), white blood cells including the differential counts are often used as the indices of immune status and stress levels in animals. A high ratio of heterophils or neutrophils to lymphocytes in blood samples is indicative of high-stress levels [116]. However, plasma immunoglobulin G (IgG) levels and white blood cells of Corriedale ewes that were deprived of water 2 and 3h after feeding were not altered. The humoral antibody response of Awassi ewes to *Salmonella enteritidis* following water restriction was found to be significantly lower and decreased by 38.50% than the control watered *ad libitum* [7]. 

The administrations of veterinary drugs or antimicrobials (e.g., gentamicin) for the treatment of several Gram-negative and some Gram-positive bacteria infections are common in animal production. Water deprivation of small ruminants that were found in water-limited areas could result in dehydration or conditions can also occur when the animals are sick and are off feed and water, thereby requiring treatment. However, changes in water compartments, kidney and liver functions [104] following water deprivation and/or restriction can predispose animals to modifications in the disposition kinetics or alter the processes determining the fate of drugs in the animal. For example gentamycinwhich is a broad spectrum aminoglycoside veterinary antibiotic, accumulates in renal proximal tubular cells following administration and its uptake is concentration-dependent [117]. In water-deprived animals, there is a possibility that uptake is enhanced by increasing the time/concentration of the drug in the plasma, thus leading to proximal tubular necrosis or nephrotoxicity [118]. Six water deprived young male Nubian goats (12–14 months), weighing 10–12 kg were used to study the pharmacokinetics of ampicillin trihydrate intravenously administered [119]. Intravenous administration (10mg/kg body weight) in the goats was studied in four stages and conditions; when (i) watered *ad lib* (ii) body weight loss averaged 7.50% following two-days water deprivation (iii) body weight loss averaged 9.80% following three-days water deprivation and (iv) body weight loss averaged 12.60% following four-days water deprivation. Each of these stages was interspersed by washout periods of free access to feed and drinking water for two, three and four weeks in stages ii, iii and iv respectively. At 7.50% body weight loss, the pharmacokinetics of the drugs (i.e., elimination half-life, volume disappearance of steady state) had very limited effect when compared to the period of hydration. However, the total body clearance of the drug was slower and significantly progressed as the per cent body weight loss increases. At 12.60% body weight loss, the volume of the central compartment (Vc) and volume of distribution at steady state (Vdss) significantly dropped. However, the author reported that the elimination half-life time of the drug (t_1/2β_) across the different periods of water deprivation was not significant. This describes the ability of the water-deprived goats to effectively cope by ensuring that the drugs progressively ‘decay’ and they are eliminated during water deprivation. In another study in which gentamycin was intravenously administered (3 mg/kg body weight) to water-deprived Nubian goats [120] and with a watering regime and body weight loss during injections similar to the study of [119], the half-life of distribution (t_1/2α_) and elimination (t_1/2β_) were not significantly affected at 7.20, 10.40 and 12.80% body weight loss. The mean plasma concentrations significantly increase as body weight continues to drop. However, the total body clearance (Cl _total_) and volume of distribution of steady state (Vdss) significantly decrease as the body weight loss increases. Oukessou and Toutain, [121] reported the limited effects on the distribution and elimination kinetics of ampicillin following a period of 72 h water deprivation. These abilities of small ruminants might account for the adaptive clearance mechanisms in animals, though to a certain level of dehydration. Body water loss is mainly from the extracellular fluid, during deprivation, especially the blood [122]. Therefore, it is expected that changes in water compartments could influence the elimination of drugs especially those with a small volume of distribution. In addition, haemoconcentration leading to a reduction in the blood flow to the kidney in dehydrated animals and the reduction of renal filtration could be responsible for the reduction in the rate of body clearance and the elimination of drugs. Impairment of renal function leading to reduced renal filtration and renal plasma flow has been confirmed in dehydrated sheep [123]. There is a need for more studies on adaptable breeds both in sheep and goats on the pharmacokinetics of drugs at different levels of water restriction and ambient temperature. 

## 6. Modulating Genes in Small Ruminants Found in Dry Areas and Future Research Gap

Drinking behaviour, maintenance of fluid, electrolyte, and homeostasis in the dehydrated animal is partly controlled by several cascades of activities and hormonal interplay with a known effect on water balance. Among them are the water-retaining hormone systems including the renin-angiotensin system, aldosterone and anti-diuretic hormone. The blocking of angiotensin II AT1 receptors (Angiotensin II receptor type 1) with losartan during 20 days water deprivation study with camels significantly enhanced the effect of dehydration on body weight reduction and increased the serum metabolites [124]. This substantiates the role of renin-angiotensin system during deprivation. The adaptation of animals to dry and water limited areas is mediated by a complex network of genes [125]. Selection for these genes using appropriate genomic tools could be the way out towards the selection of livestock to be adapted to water stress and still be able to reach an adequate level of production to support agricultural industries, especially in arid and water-limited areas. Unfortunately, selection using conventional breeding strategies is ineffective as adaptation traits are usually of low heritability (h^2^ ≤ 0.25), difficult and expensive to accurately measure. In the study of Elbeltagyet al. [126], several genetic approaches including the signature of selection (SS) analyses (identifying long conserved stretches of chromosomal regions associated with stress tolerance traits),genome-wide association (GWAS) analysis (detect genomic regions associated with stress affected physiological parameters) and 50k Illumina SNP Beadchips were deployed to investigate and compare tolerance to stress in the Egyptian desert and non-desert sheep and goats. Yang et al. [127] sequence the whole-genome of native sheep and identified four pathways and the associated genes that are responsible for the successful adaptation of Taklimakan desert sheep; arachidonic acid metabolism pathway (ANXA6 (Annexin VI), GPX3 (Glutathione Peroxidase 3), GPX7 (Glutathione Peroxidase 7), and PTGS2 (Prostanglandin-endoperoxide synthase)), renin-angiotensin system pathway (CPA3 (Carboxypeptidase A3), CPVL (Carboxypeptidase Vitellogenic Like), and ECE1 (Endothelial Converting Enzyme 1)), oxytocin signaling pathway (CALM2 (Calmodulin 2), CACNA2D1 (Calcium Voltage-Gated Channel Auxiliary Subunit Alpha2delta1), KCNJ5 (Potassium voltage-gated channel subfamily J member 5), and COX2 (cyclooxygenase2)) and pancreatic secretion pathway (RAP1A (member of RAS Oncogene Family), SLC4A4 (solute carrier family 4 member 4)). The three systems regulate water retention and re-absorption in renal cells and blood vessels in the kidney while the last one is responsible for protein and carbohydrate digestion and absorption functions. Other desert-adaptation functions that are mediated by the pathway genes include renal vasolidation, salt-water metabolism, ion transmembrane transport and bicarbonate absorption.

## 7. Conclusions

As a result of the continuous shortfall in rainfall pattern, arising from climate change, small ruminants have gained attention from scientists and communal farmers because of their ability to tolerate intermittent watering during periods of water scarcity without seriously compromising the production indices. However, there exists, differences in the level adaptation to intermittent watering across different breeds of sheep and goats. The isolation of such candidate genes using appropriate genetic tools and/or approaches, especially among adaptable breeds that have undergone natural selection for breeding and selection purposes, is a key research gap. Giving the high number of sheep and goat breeds in the world, studies investigating the potential of adaptable breed to low water intake are still few. There is a need to fully explore water tolerance capacity in adaptable breeds in the form of water restriction or deprivation and across all physiological stages.

## Figures and Tables

**Table 1:** Population size and distribution of buffalo, cattle, sheep and goat [31].

Ruminant Type/Distribution	Population Size (×10^3^)	Breed Number	Population Share (%)	Breed Share (%)	Breeds per Million
Buffalo					
Africa	0	3	0	3	0
Asia + Pacific	152,404	61	96	71	0.4
Europe	412	3	0	3	7.3
LatinAmerica + Carribean	1711	9	1	10	5.3
Near East	3998	10	1	12	2.5
NorthAmerica	0	0	0	0	0
Total	158,525	86			
Cattle					
Africa	174,556	251	13	21	1.4
Asia + Pacific	461,197	236	35	19	0.5
Europe	162,119	482	12	39	3.0
LatinAmerica + Carribean	356,069	107	27	9	0.3
Near East	71,913	86	5	7	1.2
NorthAmerica	111,481	62	8	5	0.6
Total	1,337,335	1224			
Goat					
Africa	137,104	89	19	16	0.6
Asia + Pacific	390,433	146	55	26	0.4
Europe	26,092	187	4	33	7.2
LatinAmerica + Carribean	40,752	34	6	6	0.8
Near East	114,572	94	16	16	0.8
NorthAmerica	1428	20	0	4	14.0
Total	710,381	570			
Sheep					
Africa	127,440	147	12	11	1.2
Asia + Pacific	408,098	233	39	18	0.6
Europe	185,035	629	18	48	3.4
LatinAmerica + Carribean	89,372	42	8	3	0.5
Near East	242,770	201	23	15	0.8
NorthAmerica	7891	62	1	5	7.9
Total	1,060,606	1314			

NB: Population share is the contribution of each region for a particular type of ruminant to the total world population; Breed share is the total number of breeds per fraction of world total breeds of a given ruminant inhabitants of a particular region; Breed per million is the average number of breeds in one million population number of a particular ruminant type in a given geographical area.

**Table 2:** Effect of water restriction regimen on rectal temperature in small ruminants.

Water Restriction	Specie	Average Tr (°C)		Age (Years)	Average Ambient Temperature (°C)	RH (%)	Ref.
		Water Restriction	Control				
3 days	Nubian goat	37.90	39.90 ^s^	2–3	34.8	25.5	[8]
4 days	Sheep	38.86	38.99 ^ns^	Mature	24.8	NG	[60]
4 days	Sheep	39.78	39.37 ^ns^	Mature	0.4	NG	[60]
20%, 40% less *ad lib* and alternate day	Malpura ewes	38.68, 38.80 and 38.74 respectively	38.80 ^ns^	2–4	39.86	26.03	[64]
20%, 40% less *ad lib* and alternate day *ad lib*	Malpura ewes	38.55, 38.46, and 38.47 respectively	38.53 ^ns^	2–4	32.37	36.67	[64]
Once every 3 days	Lactating Awassi ewes	39.53	39.51 ^ns^	mature	27-31	61–85	[48]
Once every 3 days	Dry Awassi ewes	39.47	39.67 ^ns^	mature	27-31	61–85	[48]
3–15 h per day	German black-head mutton sheep	38.7	39.0 ^ns^	1.8	12.7	73.5	[65]
3 h per day	German black-head mutton sheep	38.6	38.7 ^ns^	1.8	12.7	73.5	[65]
6 h on the second day	German black-head mutton sheep	38.3	38.8 ^ns^	1.8	12.7	73.5	[65]
3–15 h per day	Boer goats	37.8	38.1 ^ns^	4.3	8.9	71.0	[65]
3 h per day	Boer goats	37.8	38.1 ^ns^	4.3	8.9	71.0	[65]
6 h on the second day	Boer goats	37.3	37.7 ^ns^	4.3	8.9	71.0	[65]
50% of *ad lib*	Baladi goat	38.98	38.93 ^ns^	1.5-2	NG	NG	[66]

NG = not given; s = significant; RH = relative humidity; Tr = rectal temperature.

**Table 3:** Effect of water restriction regimen on respiratory rate in small ruminants.

Water Restriction	Specie	Average RR (Breath/Min)		Age (Years)	Ambient Temp.	RH (%)	Ref.
		Water Restriction	Control				
3–15 h per day	German black-head mutton sheep	35.9	36.7 ^s^	1.8	12.7	73.5	[65]
3 h per day	German black-head mutton sheep	34.1	36.1 ^s^	1.8	12.7	73.5	[65]
6 h on the second day	German black-head mutton sheep	32.0	25.1 ^s^	1.8	12.7	73.5	[65]
3–15 h per day	Boer Goats	20.5	20.1 ^s^	4.3	8.9	71.0	[65]
3 h per day	Boer Goats	19.1	18.4 ^s^	4.3	8.9	71.0	[65]
6 h on the second day	Boer Goats	18.7	17.6 ^s^	4.3	8.9	71.0	[65]
50% of *ad lib*	Baladi goat	34.77	36.97 ^s^	1.5-2	NG	NG	[66]
80% and 60% *ad lib*	Lacauna ewes	22.60 and 20.20 respectively	26.20 ^s^	mature	NG	NG	[49]
20% and 40% *ad lib* and alternate day	Malpura ewes	38.23, 40.40 and 37.03 respectively	46.23 ^s^	2-4	32.37	36.67	[64]
20%, 40% *ad lib* and alternate day *ad lib*	Malpura ewes	59.43, 62.87 and 60.69	67.26 ^ns^	2-4	39.86	26.03	[64]

ns = not significant; s = significant; NG = not given; RR = respiratory rate.
